# Field-Based Concurrent Validity and Test–Retest Reliability of a Portable Force Platform During IMTP and Countermovement Jump Assessments

**DOI:** 10.3390/bioengineering13060674

**Published:** 2026-06-10

**Authors:** Uğur Fidan, Mehmet Yıldız, Zeki Akyıldız, İrem Güdücü

**Affiliations:** 1 Faculty of Engineering, Biomedical Engineering, Afyon Kocatepe University, 03200 Afyonkarahisar, Türkiye; ufidan@aku.edu.tr (U.F.); irem.guducu@usr.aku.edu.tr (İ.G.); 2 Sports Science Faculty, Department of Coaching Education, Afyon Kocatepe University, 03200 Afyonkarahisar, Türkiye; mehmetyildiz@aku.edu.tr

**Keywords:** force platform validation, concurrent validity, test–retest reliability, isometric mid-thigh pull, countermovement jump

## Abstract

Portable force platforms are increasingly used for neuromuscular performance assessment in field-based environments; however, their validity may vary depending on the analyzed variable and the experimental configuration. The present study investigated the concurrent validity and test–retest reliability of a novel portable force platform (Fitforce) during commonly used static and dynamic performance assessments under field-based conditions. Thirty recreationally active male university students (age: 24.5 ± 4.1 years; height: 177.1 ± 6.18 cm; body mass: 75.38 ± 4.62 kg) performed the isometric mid-thigh pull (IMTP) and countermovement jump (CMJ) on two force platforms positioned in a stacked configuration, with the Fitforce system placed on top of a laboratory-grade reference platform (ForceDecks). Concurrent validity was evaluated using paired comparisons, intraclass correlation coefficients (ICC), coefficients of determination (R^2^), and Bland–Altman analyses. Test–retest reliability of the Fitforce system was assessed across two testing sessions conducted 24 h apart. Very high agreement was observed between systems for IMTP-derived variables (ICC = 0.95–0.98) and for CMJ propulsion-related variables, including jump height, flight time, and peak take-off force (ICC = 0.92–0.96). In contrast, peak landing force showed poor agreement across systems (ICC = −0.88, R^2^ = 0.19), with substantial systematic bias, whereas braking phase duration showed only moderate agreement (ICC = 0.50). Excellent test–retest reliability was observed across all IMTP (ICC > 0.96; CV% < 3.59) and CMJ (ICC > 0.97; CV% < 3.42) variables. Bland–Altman analyses demonstrated narrow limits of agreement for IMTP and propulsion-related CMJ variables but wide limits for landing-related force measurements. The Fitforce platform demonstrates strong concurrent agreement and excellent between-day reliability for selected IMTP and CMJ propulsion-related force–time variables under field-based conditions. However, landing-related variables should be interpreted cautiously under stacked measurement configurations due to their sensitivity to rapid impact transients and force transmission characteristics.

## 1. Introduction

In sports sciences, as in many other disciplines, technological equipment is increasingly integrated into performance assessment, monitoring, and performance enhancement processes [1]. Advances in measurement technologies have enabled more detailed and objective analyses of sporting activities, thereby substantially facilitating decision-making in training planning and performance management [2]. As a result, technological systems have profoundly transformed the sports domain by reshaping how athletes train, compete, and how athletic performance is monitored and evaluated. Data obtained from such systems are now widely used to optimize training programs, identify recovery needs, develop competitive strategies, and support the prevention and treatment of sports-related injuries [2,3].

Historically, performance testing relied primarily on manual measurements of variables such as time and distance. However, alongside technological advancements, sensor- and transducer-based automated systems have become increasingly prevalent, offering greater validity and reliability than traditional manual methods [4]. Performance tests conducted with technological equipment offer several advantages, including improved measurement accuracy, reduced testing time, and reduced practitioner workload [3]. Consequently, assessments that previously required substantial time and effort can now be completed within seconds. Some performance assessments are conducted under highly controlled laboratory conditions [5], whereas others are intended for direct implementation in field-based environments [3]. Performance tests reported in the literature are commonly grouped into force tests [6,7], power tests, speed tests (including sprint and agility), and aerobic and anaerobic endurance tests [3]. Data derived from these tests are typically obtained by measuring and processing variables such as time, distance, and force using a wide range of sensor systems, including photocell timing gates, global positioning systems (GPS), cycle ergometers, dynamometers, accelerometers, and force platforms [3]. Among these technologies, force platforms are widely regarded as the gold standard for kinetic assessment due to their ability to capture detailed force–time characteristics during both static and dynamic tasks.

Force platforms provide detailed insight into force production, impulse, and the temporal characteristics of movement and are therefore extensively used to evaluate neuromuscular performance in both athletic and clinical populations. Consequently, tests such as the isometric mid-thigh pull (IMTP) and countermovement jump (CMJ) have become central tools in performance monitoring, talent identification, and training prescription because of their strong associations with maximal strength, power output, and overall athletic performance. Despite their methodological advantages, laboratory-grade force platforms are often limited by high cost, restricted portability, and the requirement for controlled testing environments. These limitations have driven the development of portable and field-based force platform systems capable of extending force–time analysis beyond the laboratory setting. Force platforms generally consist of a rigid surface equipped with load-sensitive transducers that quantify the forces applied to the platform. These sensing systems commonly include strain gauges (load cells), accelerometers, or both [8]. Strain gauges quantify deformation induced by applied forces, whereas accelerometers measure platform acceleration. The resulting signals are subsequently transmitted to computer systems, allowing real-time or retrospective analysis of force–time characteristics [9]. Early generations of force platforms were predominantly wired and fixed systems designed for laboratory applications. In contrast, recent technological developments have led to the emergence of portable, wireless systems specifically designed for field-based performance assessment [10]. Furthermore, force platforms are available in several configurations, including single-axis, multi-component, bilateral, and wireless systems, each providing distinct measurement capabilities [8].

Modern force platform systems commonly use four or five load cells and may operate as single or multi-platform arrangements to facilitate bilateral or multi-segmental analyses. Although manufacturer-specific software varies substantially across systems, force platforms generally provide outputs for explosive force assessments, isometric force production, dynamic movement analysis, and balance evaluation. Consequently, a wide range of performance- and rehabilitation-related variables—including force, power, acceleration, flight time, contact time, eccentric and concentric phase durations, reactive strength index, neuromuscular fatigue, and postural stability—can be quantified with high precision [11]. These variables are extensively used in sports science to assess athletic performance, monitor training adaptation, support talent identification, and evaluate recovery status. Similarly, in rehabilitation and clinical settings, force platforms are widely used to reduce injury risk, support clinical assessment, monitor rehabilitation progress, and optimize treatment strategies [11]. One of the principal advantages of contemporary force platforms is their high measurement sensitivity and temporal resolution. Whereas earlier systems commonly operated at sampling frequencies near 400 Hz, modern force platforms frequently sample between 1000 and 2000 Hz, thereby enabling more accurate detection of rapid force fluctuations and transient biomechanical events. However, despite numerous studies reporting favorable validity and reliability for portable force platforms when assessing variables such as peak force, jump height, and flight time, discrepancies persist for high-impact variables, particularly peak landing force. Variability in sampling frequency, signal processing approaches, threshold definitions, and mechanical transmission pathways may substantially influence measurements obtained during rapid impact events. These observations suggest that agreement between force measurement systems may be variable-specific rather than universally consistent across all force–time characteristics. An additional methodological consideration concerns the experimental configurations commonly used in force platform validation studies. Stacked force platform arrangements, in which a portable platform is positioned directly above a laboratory-grade reference system, are commonly used to enable simultaneous data acquisition under identical movement conditions. Although this approach facilitates direct comparison between systems, it may also introduce systematic bias in variables sensitive to high-frequency impact transients due to mechanical damping, structural compliance, and energy dissipation between platforms. Consequently, interpretation of agreement for landing-related variables requires careful consideration of both sensor behavior and measurement configuration [12,13]. Although the validity and reliability of force platforms produced by established manufacturers have been extensively investigated, limited information remains available regarding newly developed systems, particularly under applied field-based conditions. Previous investigations have examined the validity and reliability of the Fitforce force platform under laboratory settings; however, its measurement properties have not yet been comprehensively evaluated in field-based environments. Establishing the validity and reliability of performance testing systems is essential to ensure that collected data accurately represent the intended performance constructs and are sufficiently consistent to detect meaningful changes over time [12,13]. Inadequate validity may lead to misleading interpretations, whereas insufficient reliability may obscure meaningful performance adaptations and compromise evidence-based decision-making [14]. Therefore, systematic evaluation of validity and reliability remains fundamental to the scientific credibility and practical applicability of emerging performance assessment technologies [15].

Therefore, the primary aim of the present study was to examine the concurrent validity and test–retest reliability of a novel portable force platform (Fitforce) for assessing selected static and dynamic force–time variables during the IMTP and CMJ under field-based conditions using a stacked force platform configuration. A secondary aim was to investigate whether agreement between systems varied with the biomechanical characteristics of the analyzed variables, particularly those involving rapid impact transients during landing. It was hypothesized that the Fitforce platform would demonstrate strong concurrent agreement and excellent between-day reliability for propulsion-related and isometric force–time variables. In contrast, variables associated with rapid impact loading, particularly peak landing force, were expected to demonstrate lower agreement due to potential mechanical damping and signal transmission effects inherent to the stacked measurement configuration.

## 2. Materials and Methods

### 2.1. Participants

Thirty recreationally active male university students (age: 24.5 ± 4.1 years; height: 177.1 ± 6.18 cm; body mass: 75.38 ± 4.62 kg) voluntarily participated in this study. All participants were regularly engaged in structured physical activity, defined as participation in sport-specific training at least three times per week, with a typical daily training duration of approximately 1–2 h. Participants were actively involved in either team-based or individual competitive sports and were assessed during their competitive season while maintaining their habitual training routines throughout the data collection period. The inclusion of recreationally active individuals with previous training experience was intended to ensure familiarity with force-based performance testing procedures while minimizing excessive inter-individual variability in neuromuscular performance characteristics. The study sample consisted exclusively of male participants to minimize the potential influence of sex-related differences in neuromuscular performance and force–time characteristics, as previously reported in the literature [16,17]. This approach was adopted to enhance internal consistency and reduce biological variability associated with sex-specific biomechanical and neuromuscular differences. However, this sampling strategy may limit the generalizability of the findings to female, clinical, or elite athletic populations. An a priori sample size estimation was conducted using G*Power software (version 3.1, Universität Düsseldorf, Düsseldorf, Germany). Based on a two-way analysis of variance (ANOVA), assuming a statistical power of 0.95, a type I error rate (α) of 0.05, and a small effect size (f = 0.05), the minimum required sample size was calculated as 20 participants. To account for a potential attrition rate of approximately 15%, the target sample size was increased to 23 participants. Ultimately, the recruitment of 30 participants ensured adequate statistical power for both validity and reliability analyses and was consistent with methodological recommendations for studies involving force measurement systems [13]. Participants were eligible for inclusion if they were healthy male athletes aged 18 to 30 years, free of any known musculoskeletal, neurological, or cardiovascular disorders, and had not undergone any surgical intervention within the preceding 6 months. In addition, all participants had prior experience with countermovement jump (CMJ) testing and were familiar with maximal-effort lower-limb performance assessments. Participants were excluded if they reported any injury, pain, or medical condition that could potentially influence force production or jumping performance during the testing period. Before participation, all individuals were fully informed about the study’s purpose, potential benefits, and possible risks associated with the experimental procedures. Ethical approval was obtained from the relevant Institutional Ethics Committee (Protocol No: 805587721/53) in accordance with the Declaration of Helsinki. Written informed consent was obtained from all participants before any testing procedures were initiated.

### 2.2. Experimental Approach and Procedures

To examine the validity and reliability of the newly developed portable force platform, both static (isometric) and dynamic (vertical jump) force–time characteristics were assessed. These testing modalities are among the most widely used and methodologically robust approaches for evaluating neuromuscular performance in sport science research [18]. Accordingly, the isometric mid-thigh pull (IMTP) and countermovement jump (CMJ) were selected as the primary assessment protocols because they provide comprehensive information regarding maximal force production, explosive lower-limb performance, and force–time characteristics commonly used in applied sport science settings. For the assessment of concurrent (criterion) validity, force–time data obtained from the newly developed portable force platform (Fitforce) were directly compared with those simultaneously collected from a laboratory-grade reference force platform (ForceDecks; VALD Performance, Brisbane Australia). The reference system has previously demonstrated high validity and reliability in force measurement applications [9,19]. To enable simultaneous data acquisition under identical movement conditions, the Fitforce platform was securely positioned atop the reference force platform in a stacked configuration. Participants performed all testing procedures while standing directly on the stacked platform arrangement. Before each testing session, both systems were zeroed and calibrated according to the manufacturer’s guidelines. Because simultaneous recordings were obtained from both platforms during the same movement task, the stacked configuration was selected to minimize inter-trial biomechanical variability and ensure synchronized acquisition conditions across systems. However, given that stacked force platform arrangements may introduce mechanical damping, structural compliance, and energy dissipation during high-impact tasks, particularly during landing phases, validity outcomes were interpreted with consideration of the potential influence of the experimental configuration on force transmission characteristics. The sampling frequency of both force platforms was standardized to 1000 Hz to ensure comparable temporal resolution and reduce potential discrepancies arising from unequal sampling rates. Simultaneous acquisition was initiated under identical testing conditions for both systems to minimize potential temporal misalignment during force–time analysis.

Before formal testing, all participants completed a standardized 10 min warm-up protocol consisting of 5 min of general dynamic activity, followed by 5 min of dynamic stretching. To ensure adequate familiarization with the testing procedures, participants subsequently performed three submaximal squat movements and three practice countermovement jumps. In addition, all participants were verbally instructed regarding the correct execution of each testing procedure before data collection commenced. Following familiarization, participants performed the CMJ and IMTP protocols on the stacked force platform system while force–time variables from both platforms were simultaneously recorded for concurrent validity analysis. Test–retest reliability was evaluated using repeated measurements obtained exclusively from the Fitforce force platform across two separate testing sessions conducted 24 h apart under consistent environmental and testing conditions. During each session, participants performed three maximal CMJ and IMTP trials, with standardized recovery periods provided between attempts to minimize fatigue-related effects and ensure maximal voluntary effort throughout testing.

### 2.3. Testing Protocols

#### 2.3.1. IMTP Protocol

The isometric mid-thigh pull (IMTP) test was employed to assess maximal static force production of the lower limbs. The IMTP was selected because it is widely recognized as a reliable and biomechanically robust method for evaluating maximal force-generating capacity and neuromuscular performance in both research and applied sport settings [16,18]. In addition, IMTP-derived force–time variables are frequently used in methodological investigations examining the validity and reliability of force measurement systems.

All IMTP measurements were performed using a Smith machine to constrain the bar path and minimize movement variability between trials and participants. Force data were collected using a force platform system positioned directly beneath the Smith machine and in full contact with the ground. This experimental configuration has previously been shown to reduce potential horizontal and vertical force losses during data acquisition, thereby improving signal quality and measurement consistency [17]. Compared with free-bar IMTP assessments, Smith machine–based protocols provide greater procedural standardization by reducing technical variability and limiting extraneous movement artifacts that may influence force measurements [16,17]. Such standardization is particularly important in studies evaluating the validity and reliability of force measurement systems, as it minimizes measurement variability unrelated to the force platform’s performance. Consequently, Smith machine–based IMTP protocols are widely accepted as valid and reliable approaches for assessing maximal lower-limb force production [16]. Before data collection, all participants completed the standardized warm-up and familiarization procedures described previously. The IMTP testing position was subsequently individualized for each participant based on their anthropometric characteristics to ensure consistent biomechanical alignment across testing sessions. Participants were positioned beneath the fixed bar with knee joint angles of approximately 125–145° and hip joint angles of approximately 140–150°. These joint angle ranges were selected according to established methodological recommendations to optimize maximal force production and improve test–retest reliability [20]. Bar height and body positioning were standardized for each participant and remained unchanged throughout all testing sessions.

**Figure 1:**
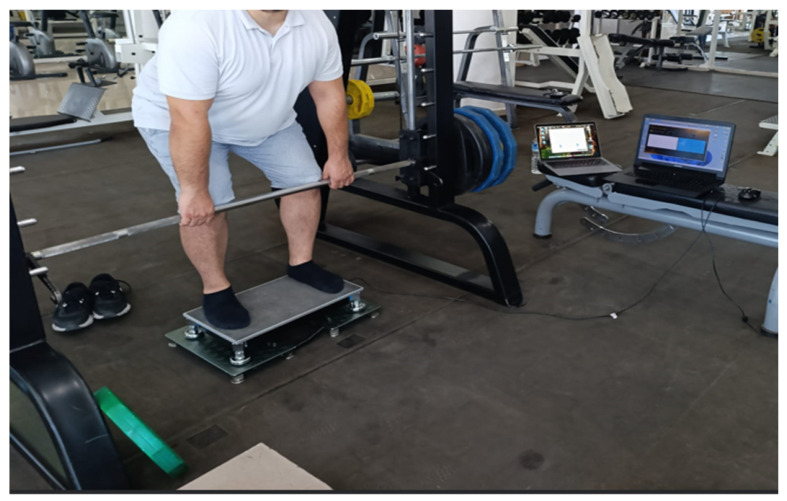
IMTP testing protocol.

During each IMTP trial (Figure 1), participants were instructed to pull vertically against the immovable bar “as fast and as forcefully as possible” while maintaining maximal voluntary effort for approximately 3–5 s. Standardized verbal encouragement was provided during all trials to promote maximal effort and reduce motivational variability between attempts. Throughout each contraction, participants were instructed to maintain full heel contact with the platform and preserve a stable trunk posture to ensure consistent force application. Each participant performed two to three maximal IMTP trials, separated by standardized recovery intervals of 2–3 min to minimize fatigue-related effects [16,18]. The investigators visually monitored trials to identify any technical errors, countermovement behavior, excessive postural deviation, or loss of heel contact that could compromise data quality. Trials that did not meet the predefined procedural criteria were discarded and repeated. For subsequent statistical analyses, the highest peak force (PF) value and selected force–time variables derived from valid IMTP trials were retained for evaluation, consistent with previous methodological studies investigating maximal force production characteristics [16,18].

#### 2.3.2. Countermovement Jump (CMJ) Protocol

**Figure 2:**
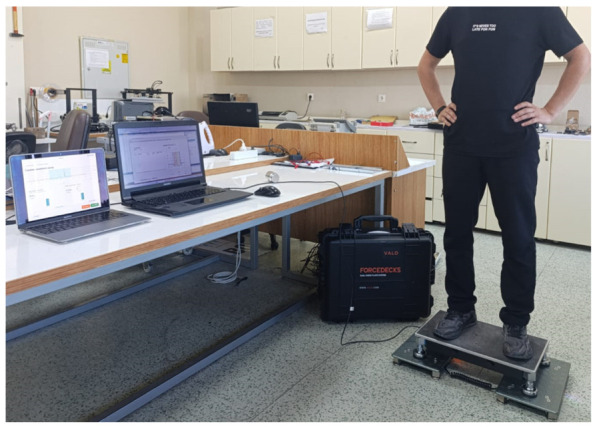
CMJ testing protocol.

The countermovement jump (CMJ) was employed as a dynamic assessment to evaluate lower-limb explosive performance and to examine the concurrent validity and test–retest reliability of the force platform systems. The CMJ is widely recognized as a gold-standard assessment of neuromuscular function because of its high ecological validity, sensitivity to performance-related changes, and strong reliability across a broad range of athletic populations [21]. Furthermore, CMJ-derived force–time variables are frequently used in methodological investigations aimed at validating force measurement technologies [9,22]. All CMJ trials were performed on the force platform system, with participants positioned upright, feet approximately shoulder-width apart, and hands fixed on the hips throughout the movement to eliminate the potential influence of arm swing on jump mechanics and force production. Participants were instructed to perform a rapid downward countermovement to a self-selected depth, immediately followed by a maximal vertical jump with emphasis placed on achieving maximal jump height. The use of a self-selected countermovement depth was intended to preserve natural jumping mechanics and reduce movement constraints that could alter force–time behavior. Standardized verbal encouragement was provided during all trials to ensure maximal voluntary effort and reduce inter-trial variability, thereby improving the reliability of CMJ-derived outcome measures [16]. Before experimental testing (Figure 2), all participants completed the standardized warm-up and familiarization procedures described previously, including several submaximal and maximal practice jumps to minimize potential learning effects and ensure consistent movement execution. During data collection, each participant performed two to three maximal CMJ trials, with standardized recovery intervals of 2–3 min between attempts to minimize fatigue-related performance decrements. All trials were visually monitored by the investigators to ensure correct movement execution and to identify potential technical errors, including loss of hand position, excessive countermovement asymmetry, or non-standard movement patterns. Trials deemed technically invalid were discarded and repeated. Consistent with several previous methodological investigations, the best-performing trial, defined as the one producing the greatest jump height, was retained for subsequent statistical analyses, as this approach has been shown to provide a reliable representation of maximal explosive performance capacity [23]. However, given the potential influence of trial selection strategies on reliability and agreement outcomes, this methodological consideration was taken into account in the interpretation of the findings.

Force–time data were sampled at a high frequency (≥1000 Hz) and subsequently processed to derive key CMJ variables, including jump height (cm), flight time (ms), peak take-off force (N), peak landing force (N), and braking phase duration (ms). Jump height was calculated using force–time integration procedures, which have previously demonstrated greater accuracy than flight-time-based estimation methods when force platform data are available [21]. In addition to propulsion-related variables, landing-related force characteristics were also analyzed to examine system behavior during rapid impact transients. Because landing phases involve high-frequency force fluctuations and rapid loading rates, these variables may be particularly sensitive to signal-processing approaches, threshold definitions, and mechanical transmission characteristics of stacked force platforms. Consequently, landing-related outcomes were interpreted with consideration of the potential influence of measurement configuration on force transmission behavior. Collectively, this standardized CMJ protocol provided high measurement consistency and was considered appropriate for evaluating the concurrent validity and test–retest reliability of the force platform systems under field-based testing conditions.

### 2.4. Data Processing and Variable Definitions

All force–time data obtained from both force platforms were exported and processed offline using manufacturer-specific software and standardized analytical procedures. To ensure methodological consistency during the concurrent validity assessment, identical processing criteria and event-detection procedures were applied to both systems whenever possible. All analyses were performed using the vertical ground reaction force (vGRF) signal obtained from each platform. Before variable extraction, all force–time signals were visually inspected to identify potential artifacts, signal irregularities, baseline drift, or offset-related abnormalities that could compromise data quality. System zeroing procedures were performed before each trial according to the manufacturer’s guidelines. Body weight was determined during a quiet standing phase immediately preceding each trial and subsequently subtracted from the force signal to facilitate accurate phase detection and reliable calculation of force-related variables.

Both force platforms sampled data at 1000 Hz, thereby providing high temporal resolution for detecting rapid force fluctuations and transient biomechanical events. Force signals were analyzed in their raw form without additional low-pass filtering. This methodological decision was made to avoid potential attenuation, smoothing, or temporal distortion of high-frequency force components, particularly during rapid impact events such as landing, which represented a primary focus of the present investigation. Similar approaches have previously been reported in force platform validation studies examining transient force characteristics [9,11,21]. Nevertheless, it is acknowledged that unfiltered force signals may be more susceptible to high-frequency noise and signal variability, particularly during impact-related phases involving rapid loading rates. Consequently, landing-related variables were interpreted with caution, and the potential influence of signal noise and mechanical transmission characteristics on high-impact variables was considered during data interpretation.

For the isometric mid-thigh pull (IMTP), force–time curves were initially examined to confirm the absence of countermovement behavior or excessive pre-tension before force onset. Force onset was defined as the point at which vertical ground reaction force exceeded body weight by 5% for a continuous duration of at least 30 ms. This threshold-based criterion was selected in accordance with previously established methodological recommendations for IMTP analysis and to improve consistency in onset detection across trials [20]. Peak force (PF) was subsequently identified as the maximum vertical force recorded during the isometric contraction phase. For each participant, only the highest PF value obtained across valid trials was retained for statistical analysis. For the countermovement jump (CMJ), force–time data were segmented into discrete movement phases using threshold-based event detection procedures. Movement onset was identified as the point at which vertical force deviated from body weight by more than 5% for at least 30 ms, indicating initiation of the countermovement phase. Take-off was defined as the instant at which vertical force fell below a threshold of 20 N, representing loss of contact with the force platform. Landing was subsequently identified when the vertical force exceeded 20 N following the flight phase. These threshold values were selected based on previously published force platform methodologies and widely accepted CMJ processing procedures [21,24]. Jump height was calculated using force–time integration methods. Vertical velocity was derived through numerical integration of the net vertical force divided by body mass, and jump height was subsequently calculated from take-off velocity. Compared with flight-time-based estimation procedures, force–time integration methods have demonstrated superior accuracy when force platform data are available [21,24]. Before integration, baseline offset correction was applied to minimize potential integration drift and improve velocity calculation stability.

In addition to jump height, several phase-specific force–time variables were extracted from the CMJ trials. Peak take-off force was defined as the maximum vertical force recorded during the concentric propulsion phase immediately preceding take-off. Peak landing force was defined as the maximum vertical force recorded during the initial ground contact following the flight phase. Braking phase duration was defined as the time interval between the onset of eccentric braking, corresponding to the minimum center-of-mass velocity, and the instant of zero vertical velocity immediately preceding the concentric propulsion phase. Because the present study employed a stacked force platform configuration for concurrent validity assessment, particular attention was paid to variables associated with rapid impact transients. Mechanical damping, structural compliance, and energy dissipation between stacked platforms may alter the transmission of high-frequency force components, particularly during landing-related phases. Therefore, although peak landing force was included to evaluate system behavior during high-impact loading conditions, this variable was interpreted cautiously during subsequent validity analyses. Accordingly, validity outcomes associated with landing-related variables should be considered configuration-dependent rather than universally generalizable across independent force platform arrangements.

### 2.5. Statistical Analysis

Before statistical analysis, the distributional properties of all variables were examined to assess the assumption of normality. Normality was evaluated using the Shapiro–Wilk test, along with visual inspection of skewness, kurtosis, and distribution plots. Because all variables demonstrated approximately normal distributions, parametric statistical procedures were subsequently applied. Concurrent validity between the Fitforce force platform and the reference system was assessed using paired-sample statistical approaches because measurements were obtained simultaneously from the same participants under identical testing conditions. Agreement between systems was quantified using intraclass correlation coefficients (ICC), coefficients of determination (R^2^), and Bland–Altman analyses. Intraclass correlation coefficients were calculated using a two-way mixed-effects model with absolute agreement for single measurements [ICC(3,1)], which is considered appropriate for assessing agreement between repeated measurements obtained from fixed measurement systems. To improve the interpretability of agreement analyses, ICC values were additionally reported with corresponding 95% confidence intervals (95% CI). Differences between platforms were examined using paired-samples *t*-tests, and effect sizes were calculated using Cohen’s d to quantify the magnitude of between-system differences. Effect sizes were interpreted according to conventional thresholds: trivial (<0.20), small (0.20–0.49), moderate (0.50–0.79), and large (≥0.80). Bland–Altman plots were constructed to evaluate systematic bias and limits of agreement between measurement systems. In addition to visual inspection, mean bias and 95% limits of agreement (LOA) were calculated for each variable. Because variables involving larger force magnitudes may exhibit proportional error, Bland–Altman distributions were also visually inspected for evidence of heteroscedasticity and variance scaling across measurement ranges. Test–retest reliability of the Fitforce force platform was evaluated using repeated measurements obtained across two separate testing sessions. Reliability indices included the intraclass correlation coefficient (ICC), coefficient of variation (CV%), standard error of measurement (SEM), and minimal detectable change at the 95% confidence level (MDC95). The coefficient of variation was calculated using the formula:

$$CV\left(\%\right)=\frac{SD}{Mean}\times 100$$

The standard error of measurement (SEM) was calculated as:

$$SEM=SD\times \sqrt{1-ICC}$$

The minimal detectable change at the 95% confidence level (MDC95) was calculated as:

$${MDC}_{95}=SEM\times 1.96\times \sqrt{2}$$

These additional reliability metrics were included to provide a more comprehensive interpretation of measurement consistency and practical sensitivity beyond correlation-based indices alone. ICC values were interpreted according to the following thresholds: extremely high (0.90–0.99), very high (0.75–0.90), high (0.50–0.75), moderate (0.20–0.50), and low (<0.20) [13,14,15]. Reliability was considered acceptable when the CV% was <8% and the ICC was ≥0.80 [13]. However, high ICC values were not interpreted as evidence of direct interchangeability between systems in isolation, and validity outcomes were interpreted in conjunction with systematic bias, limits of agreement, and variable-specific measurement behavior. All statistical analyses were performed using JASP software (version 0.19.3; University of Amsterdam, Amsterdam, The Netherlands). Data are presented as mean ± standard deviation (SD). Statistical significance was established at *p* < 0.05 for all analyses.

## 3. Results

### 3.1. Concurrent Validity for IMTP Parameters

**Figure 3:**
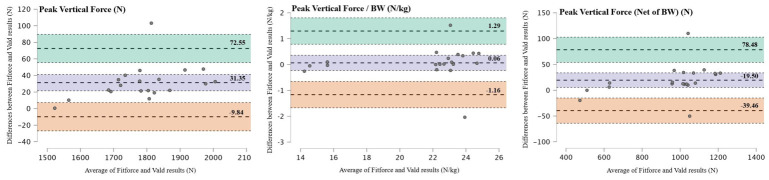
The validity data for the IMTP. The 95% limits of agreement are shown as continuous lines above and below the mean difference, with the dashed line indicating the mean difference.

Concurrent validity analysis demonstrated very high agreement between the Fitforce and ForceDecks systems for all IMTP-derived variables (Table 1). Intraclass correlation coefficients ranged from 0.95 to 0.98, indicating excellent agreement between the two measurement systems across all analyzed IMTP variables. Strong linear associations were also observed, with coefficients of determination (R^2^) ranging from 0.96 to 0.98, suggesting a high degree of shared variance between systems. For peak vertical force, the mean value recorded by the Fitforce platform was marginally lower than that obtained from the ForceDecks system (mean difference = −31.35 N). However, this difference was not statistically significant (*p* = 0.43) and demonstrated only a small effect size (Cohen’s d = −0.25). Similarly, peak vertical force normalized to body mass demonstrated negligible between-system differences (mean difference = −0.06 N·kg^−1^, *p* = 0.95, Cohen’s d = −0.019), accompanied by an excellent ICC value of 0.98. Peak vertical force values calculated net of body mass also demonstrated excellent agreement between systems (ICC = 0.98, R^2^ = 0.98). The mean difference for this variable was −19.50 N, with no statistically significant difference detected between platforms (*p* = 0.78) and only a trivial effect size (Cohen’s d = −0.089). Bland–Altman analyses further supported these findings, demonstrating that differences between Fitforce and ForceDecks measurements for all IMTP variables were randomly distributed around the mean bias and remained within relatively narrow limits of agreement (Figure 3). Visual inspection of the Bland–Altman plots revealed no substantial evidence of proportional bias or marked heteroscedasticity across the measurement range, suggesting relatively stable agreement between systems for IMTP-derived force variables. Collectively, these findings indicate that the Fitforce platform demonstrates strong concurrent agreement with the laboratory-grade reference system for IMTP-derived force variables under the stacked measurement configuration employed in the present study.

### 3.2. Concurrent Validity for CMJ Parameters

**Figure 4:**
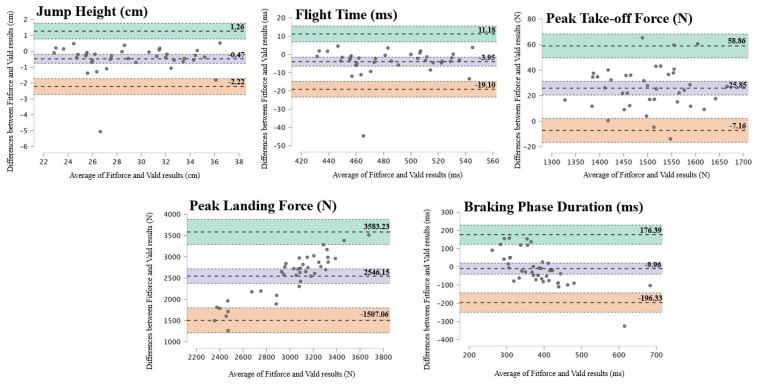
The validity data for the CMJ. The 95% limits of agreement are shown as continuous lines above and below the mean difference, with the dashed line indicating the mean difference.

Concurrent validity analyses for CMJ-derived variables are presented in Table 2. Jump height and flight time demonstrated very high agreement between the Fitforce and ForceDecks systems, with intraclass correlation coefficients of 0.96 for both variables and strong coefficients of determination (R^2^ = 0.94). Mean differences between systems were minor and not statistically significant for jump height (0.47 cm, *p* = 0.58, Cohen’s d = 0.12) or flight time (3.95 ms, *p* = 0.59, Cohen’s d = 0.12), indicating minimal systematic bias between platforms for these propulsion-related variables. Peak take-off force also demonstrated high agreement across systems (ICC = 0.92, R^2^ = 0.95). Although the Fitforce platform recorded slightly lower values compared with the ForceDecks system (mean difference = −25.85 N), this difference did not reach statistical significance (*p* = 0.14). It was associated with only a small-to-moderate effect size (Cohen’s d = −0.33). Bland–Altman analyses for jump height, flight time, and peak take-off force revealed relatively narrow limits of agreement and no substantial evidence of proportional bias across the measurement range (Figure 4). In contrast, peak landing force demonstrated poor agreement between the two systems, with a negative intraclass correlation coefficient (ICC = −0.88) and a low coefficient of determination (R^2^ = 0.19). Substantially higher peak landing force values were recorded by the ForceDecks system compared with Fitforce, resulting in a large and statistically significant mean difference (−2546.15 N, *p* < 0.001) accompanied by a very large effect size (Cohen’s d = −6.04). Bland–Altman analysis further demonstrated wide limits of agreement and substantial systematic bias for this variable, indicating a lack of interchangeability between systems for landing-related force measurements under the present experimental conditions. The braking phase duration showed only moderate agreement between systems (ICC = 0.50, R^2^ = 0.37). The mean difference between platforms was 9.96 ms, which was not statistically significant (*p* = 0.63) and demonstrated a trivial effect size (Cohen’s d = 0.11). Visual inspection of the Bland–Altman plot for braking phase duration showed greater variability across observations than for propulsion-related variables.

Collectively, these findings suggest that agreement between the Fitforce and reference systems was variable-specific rather than uniform across all CMJ-derived force–time characteristics. Variables associated with propulsion-related phases demonstrated acceptable concurrent agreement. In contrast, variables associated with rapid impact transients, particularly peak landing force, showed substantial disagreement under the stacked measurement configuration used in the present study.

### 3.3. Test–Retest Reliability for IMTP Parameters

The test–retest reliability outcomes for IMTP-derived variables obtained from the Fitforce force platform are presented in Table 2. Excellent between-day reliability was observed across all analyzed IMTP parameters, indicating high measurement consistency across testing sessions. Peak vertical force demonstrated a very high intraclass correlation coefficient (ICC = 0.96) accompanied by a low coefficient of variation (CV = 1.14%), indicating minimal measurement variability across repeated assessments. Similarly, peak vertical force normalized to body mass demonstrated near-perfect reliability (ICC = 0.99) with low variability (CV = 2.53%). Peak vertical force, calculated net of body mass, also exhibited excellent reliability (ICC = 0.98) and low variability (CV = 3.59%). Mean values obtained during the test and retest sessions were nearly identical for all IMTP-derived variables, and corresponding effect sizes were trivial (Cohen’s d ≤ 0.011), suggesting the absence of substantial systematic bias between repeated measurements. In addition, SEM and MDC95 values remained low across all variables, further supporting the stability and practical consistency of repeated IMTP measurements obtained using the Fitforce platform.

**Figure 5:**
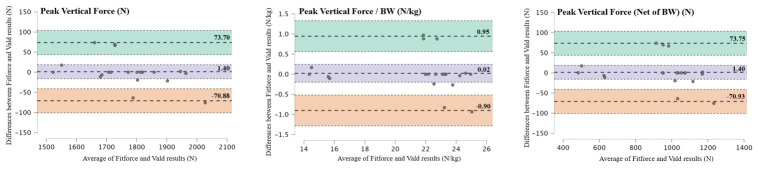
The reliability data for IMTP. The 95% limits of agreement are shown as continuous lines above and below the mean difference, with the dashed line indicating the mean difference.

Bland–Altman analyses further supported these findings by demonstrating a random distribution of differences around the mean bias together with relatively narrow limits of agreement for all IMTP variables (Figure 5). Visual inspection of the Bland–Altman plots revealed no meaningful proportional bias or heteroscedasticity across the measurement range. Collectively, these findings indicate that the Fitforce force platform demonstrates excellent between-day test–retest reliability for IMTP-derived force variables under the standardized testing conditions employed in the present study.

### 3.4. Test–Retest Reliability for CMJ Parameters

The test–retest reliability outcomes for CMJ-derived variables obtained from the Fitforce force platform are presented in Table 2. Excellent between-day reliability was observed across all analyzed CMJ performance variables, indicating high measurement consistency across repeated testing sessions. Jump height demonstrated a very high intraclass correlation coefficient (ICC = 0.98) accompanied by a low coefficient of variation (CV = 1.51%). Similarly, flight time demonstrated excellent reliability (ICC = 0.98) with very low variability (CV = 0.75%). Peak take-off force exhibited near-perfect reliability (ICC = 0.99) together with the lowest variability among CMJ variables (CV = 0.59%), indicating highly stable propulsion-related force measurements across sessions.

Peak landing force also demonstrated excellent between-day reliability (ICC = 0.97) with low variability (CV = 0.89%). However, despite this high within-device consistency, the concurrent validity analysis demonstrated poor agreement between systems for this variable. Therefore, these findings suggest that excellent reliability should not be interpreted as evidence of interchangeability or criterion validity for landing-related force measurements under the stacked configuration employed in the present study. Braking phase duration demonstrated near-perfect reliability (ICC = 0.99), although this variable exhibited slightly greater variability (CV = 3.42%) compared with the remaining CMJ-derived variables. Across all CMJ parameters, mean differences between test and retest sessions were negligible, with trivial effect sizes (Cohen’s d ranging from −0.078 to −0.006), indicating minimal systematic bias between repeated measurements.

**Figure 6:**
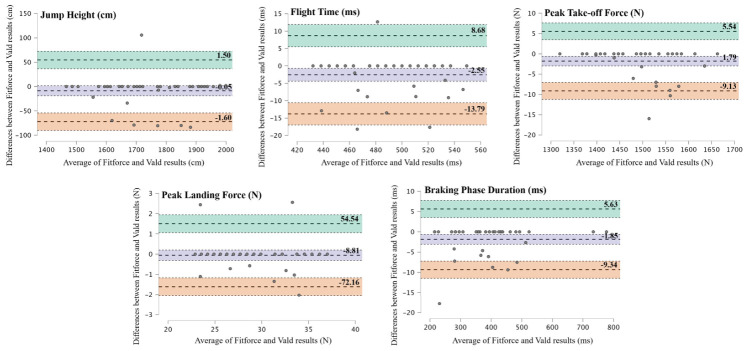
The reliability data for CMJ. The 95% limits of agreement are shown as continuous lines above and below the mean difference, with the dashed line indicating the mean difference.

Additional reliability metrics, including SEM and MDC95, further supported the consistency of repeated CMJ measurements obtained using the Fitforce platform. Bland–Altman analyses demonstrated narrow limits of agreement and minimal systematic bias across all CMJ-derived variables (Figure 6). Visual inspection of the Bland–Altman distributions revealed no substantial evidence of proportional bias or marked heteroscedasticity. Collectively, these findings confirm that the Fitforce force platform demonstrates excellent between-day reliability for CMJ-derived force–time variables under standardized testing conditions. Nevertheless, the discrepancy observed between reliability and concurrent validity outcomes for peak landing force highlights the importance of distinguishing within-device consistency from between-system agreement when interpreting force platform validation data.

## 4. Discussion

The present study investigated the concurrent validity and test–retest reliability of the Fitforce portable force platform during commonly used static and dynamic neuromuscular performance assessments under field-based conditions. The primary findings demonstrated that the Fitforce system showed strong concurrent agreement and excellent between-day reliability for most IMTP-derived and CMJ propulsion-related force–time variables. In contrast, landing-related variables, particularly peak landing force, demonstrated poor agreement between systems despite high within-device reliability. These findings suggest that the validity of portable force platforms may be variable-specific and strongly influenced by both biomechanical task characteristics and experimental measurement configuration.

The concurrent validity analyses demonstrated very high agreement between the Fitforce and reference force platform systems for IMTP-derived peak force and for CMJ propulsion-related variables, including jump height, flight time, and peak take-off force. The observed ICC values (>0.92) and narrow Bland–Altman limits of agreement indicate that the Fitforce platform can provide stable and practically comparable measurements for variables primarily associated with controlled force production and propulsion mechanics. These findings are consistent with previous investigations reporting favorable agreement between portable and laboratory-grade force platform systems for propulsion-related force–time characteristics [9,21,22]. Furthermore, the excellent test–retest reliability observed across all IMTP and CMJ variables suggests that the Fitforce system provides highly consistent measurements across repeated testing sessions, supporting its applicability for longitudinal athlete monitoring and field-based performance assessment.

However, an important finding of the present study was the poor agreement observed for peak landing force measurements. Unlike propulsion-related variables, peak landing force demonstrated substantial systematic bias and a negative ICC value between systems. Importantly, although test–retest reliability for this variable remained high within the Fitforce system, concurrent agreement between platforms was poor, highlighting the important distinction between reliability and validity. Reliability reflects the consistency of repeated measurements within the same system, whereas validity and agreement concern the extent to which measurements correspond to those obtained from a reference method. Therefore, high reliability does not necessarily imply interchangeability between systems. Negative ICC values may occur when between-system disagreement exceeds between-subject variability, indicating poor absolute agreement rather than instability of repeated measurements.

Several methodological and biomechanical factors may explain the reduced agreement observed for landing-related variables. The present study employed a stacked force platform configuration to enable simultaneous data acquisition under identical movement conditions. Although this approach minimizes inter-trial biomechanical variability and allows direct synchronization of measurements, it may also introduce mechanical damping, structural compliance, and energy dissipation during high-impact tasks. From a mechanical perspective, the stacked arrangement may behave as a coupled mass–spring–damper system during landing, potentially attenuating high-frequency impact transients before they reach the lower reference platform. Consequently, discrepancies observed in landing force measurements may reflect not only sensor-related differences but also alterations in the force transmission characteristics inherent to the experimental configuration. Therefore, the present findings should be interpreted specifically within the context of stacked measurement conditions and should not be generalized to independent non-stacked force platform setups.

The present findings further suggest that force platform agreement may depend on the biomechanical characteristics of the analyzed variable. Variables such as IMTP peak force, jump height, and take-off force are primarily influenced by relatively controlled force production patterns and lower-frequency force characteristics. In contrast, landing-related variables involve extremely rapid force transients, high loading rates, and substantial high-frequency signal content. These characteristics may increase sensitivity to small differences in signal acquisition, threshold detection, temporal synchronization, and mechanical transmission behavior between systems. Consequently, landing-related metrics may represent a more demanding validation challenge than propulsion-related variables in portable force platform research.

An additional methodological consideration involves signal processing procedures. In the present study, force signals were analyzed in the raw form, without low-pass filtering, to avoid attenuation of rapid impact-related force components during landing. Although this approach preserved high-frequency signal content, the absence of filtering may also have increased susceptibility to noise and signal variability, particularly for variables characterized by rapid force fluctuations. Previous studies have demonstrated that filtering strategies can substantially influence peak landing force measurements and other transient force characteristics. Therefore, future investigations should examine the effects of different filtering approaches and cutoff frequencies on the agreement of portable force platforms, particularly for landing-related variables involving high-frequency impact events.

The current study was designed as an applied field-based validation investigation rather than a laboratory-grade metrological characterization of the instrumentation system. Consequently, analyses such as dynamic calibration procedures, uncertainty budgeting, sensor-level hysteresis assessment, and cross-axis sensitivity evaluation were beyond the scope of the present study. Instead, the primary objective was to determine whether the Fitforce platform could provide practically acceptable agreement and reliable measurements under ecologically relevant testing conditions commonly encountered in sports performance settings. Nevertheless, future studies incorporating instrumentation-level metrological analyses and independent calibration procedures would further strengthen the understanding of portable force platform measurement behavior.

Several additional methodological considerations should also be acknowledged. First, the study included only recreationally active male participants, which may limit the generalizability of the findings to female, clinical, youth, or elite athletic populations. Second, using the best-performing trial rather than mean trial values may have influenced reliability and agreement outcomes. Although best-trial selection is commonly used in maximal performance testing and has been recommended in previous CMJ and IMTP investigations, mean-based approaches may provide greater stability for reliability analyses and should be explored in future research. Third, movement execution was standardized using visual monitoring procedures rather than full kinematic motion capture analysis. Future investigations incorporating synchronized biomechanical analyses may provide additional insight into the interaction between movement mechanics and force transmission behavior during stacked measurements.

Despite these limitations, the present study provides important practical implications for field-based neuromuscular performance assessment. The findings indicate that the Fitforce platform demonstrates strong concurrent agreement and excellent between-day reliability for selected IMTP and CMJ propulsion-related variables under applied testing conditions. Therefore, the system may represent a practical and portable alternative for monitoring maximal strength and explosive performance outside laboratory environments. However, practitioners should interpret landing-related variables cautiously when stacked measurement configurations are used, as these variables appear particularly sensitive to rapid impact transients and force transmission effects. Future research should further investigate the influence of experimental configuration, signal filtering strategies, and independent calibration procedures on the agreement of portable force platforms during high-impact biomechanical tasks.

## 5. Conclusions

The present study demonstrated that the Fitforce force platform system provides strong concurrent agreement and excellent test–retest reliability for selected force–time variables derived from the isometric mid-thigh pull (IMTP) and countermovement jump (CMJ) under field-based testing conditions. Very high agreement with a laboratory-grade reference system was observed for IMTP-derived variables and for CMJ propulsion-related metrics, including jump height, flight time, and peak take-off force. These findings support the use of the Fitforce system as a practical and reliable tool for field-based assessment of neuromuscular performance. In contrast, peak landing force showed poor agreement across systems, with substantial systematic bias and wide limits of agreement. The present findings suggest that this discrepancy was likely influenced by the stacked force platform configuration and the associated mechanical damping and force transmission characteristics during rapid impact events rather than by instability of the Fitforce platform itself. Importantly, despite limited between-system agreement, the Fitforce system demonstrated excellent within-device reliability for landing-related variables, indicating stable and consistent repeated measurements over time.

Collectively, these findings emphasize the importance of distinguishing between reliability and concurrent validity when evaluating force platform performance. High within-device reliability does not necessarily indicate direct interchangeability between independent measurement systems, particularly for variables involving rapid impact transients. Accordingly, validity outcomes should be interpreted as variable-specific and configuration-dependent rather than universally transferable across all force–time characteristics and testing arrangements. From an applied perspective, the Fitforce force platform appears suitable for field-based monitoring of IMTP-derived variables and CMJ propulsion-related performance metrics within standardized testing environments. However, landing-related outcomes should be interpreted cautiously, particularly when comparisons are made across different force platform systems or experimental configurations. Future research should further investigate independent non-stacked measurement arrangements, alternative signal-processing strategies, and dynamic calibration procedures to better characterize the behavior of portable force platform systems during high-frequency impact events.

## Figures and Tables

**Table 1:** Concurrent agreement and validity outcomes for IMTP- and CMJ-derived force–time variables obtained from the Fitforce and ForceDecks systems under stacked measurement conditions.

Test	Variable	Fitforce Mean ± SD	ForceDecks Mean ± SD	Mean Bias (Fitforce − ForceDecks)	ICC (95% CI)	R^2^	Bland–Altman Bias ± LoA	*p*-Value	Cohen’s d	Agreement Interpretation
IMTP	Peak Vertical Force (N)	1774.04 ± 121.23	1805.40 ± 129.41	−31.35	0.95 (0.89–0.98)	0.97	Narrow LoA	0.43	−0.25	Very High Agreement
IMTP	Peak Vertical Force/BM (N·kg^−1^)	21.58 ± 3.47	21.64 ± 3.53	−0.06	0.98 (0.95–0.99)	0.96	Narrow LoA	0.95	−0.02	Very High Agreement
IMTP	Peak Vertical Force (Net BM) (N)	950.49 ± 214.39	970.00 ± 255.69	−19.50	0.98 (0.95–0.99)	0.98	Narrow LoA	0.78	−0.09	Very High Agreement
CMJ	Jump Height (cm)	29.30 ± 3.92	28.82 ± 3.93	0.47	0.96 (0.90–0.98)	0.94	Narrow LoA	0.58	0.12	Very High Agreement
CMJ	Flight Time (ms)	487.80 ± 32.64	483.85 ± 32.96	3.95	0.96 (0.90–0.98)	0.94	Narrow LoA	0.59	0.12	Very High Agreement
CMJ	Peak Take-off Force (N)	1488.42 ± 78.65	1514.27 ± 79.17	−25.85	0.92 (0.83–0.96)	0.95	Moderate LoA	0.14	−0.33	High Agreement
CMJ	Peak Landing Force (N)	1734.89 ± 138.31	4281.05 ± 580.09	−2546.15	−0.88 (−0.97 to −0.57)	0.19	Wide LoA	<0.001	−6.04	Poor Agreement
CMJ	Braking Phase Duration (ms)	388.11 ± 120.02	378.15 ± 59.26	9.96	0.50 (0.13–0.75)	0.37	Wide LoA	0.63	0.11	Moderate Agreement

SD: standard deviation, ICC: intraclass correlation coefficient, IMTP: the isometric mid-thigh pull, CMJ: countermovement jump.

**Table 2:** Test- retest reliability of the Fitforce force platform.

Parameters	Test Mean ± SD	Retest Mean ± SD	ICC (95% CI)	CV (%)	SEM	MDC95	Cohen’s d	Reliability Interpretation
IMTP								
Peak Vertical Force (N)	1774.04 ± 121.23	1772.63 ± 137.71	0.96 (0.93–0.98)	1.14	25.11	69.53	0.011	Excellent
Peak Vertical Force/BM (N·kg^−1^)	21.58 ± 3.47	21.56 ± 3.53	0.99 (0.98–0.99)	2.53	0.55	1.53	0.006	Excellent
Peak Vertical Force (Net BM) (N)	950.49 ± 214.39	950.09 ± 222.65	0.98 (0.97–0.99)	3.59	7.89	21.85	0.006	Excellent
CMJ								
Jump Height (cm)	29.30 ± 3.92	29.36 ± 4.03	0.98 (0.97–0.99)	1.51	0.47	1.31	−0.014	Excellent
Flight Time (ms)	487.80 ± 32.64	490.36 ± 33.06	0.98 (0.96–0.99)	0.75	2.46	6.81	−0.078	Excellent
Peak Take-off Force (N)	1488.42 ± 78.65	1490.21 ± 79.74	0.99 (0.98–0.99)	0.59	4.21	11.67	−0.006	Excellent
Peak Landing Force (N)	1734.89 ± 138.31	1743.71 ± 139.71	0.97 (0.95–0.98)	0.89	5.45	15.10	−0.063	Excellent
Braking Phase Duration (ms)	388.11 ± 120.02	389.96 ± 119.50	0.99 (0.98–0.99)	3.42	4.97	13.78	−0.015	Excellent

SD: standard deviation, ICC: intraclass correlation coefficient, CV: coefficient of variation, SEM: standart error of measurement, IMTP: the isometric mid-thigh pull, CMJ: countermovement jump.

## Data Availability

Data are available to the corresponding author upon reasonable request.

## Abbreviations

The following abbreviations are used in this manuscript:

ANOVA	Analysis of Variance
BM	Body Mass
CMJ	Countermovement Jump
CV (%)	Coefficient of Variation
GPS	Global Positioning System
ICC	Intraclass Correlation Coefficient
IMTP	Isometric Mid-Thigh Pull
PF	Peak Force
R^2^	Coefficient of Determination
SD	Standard Deviation
vGRF	Vertical Ground Reaction Force
